# Systematic review and meta-analysis of clinical outcomes of COVID-19 patients undergoing gastrointestinal endoscopy

**DOI:** 10.1177/17562848211042185

**Published:** 2021-08-30

**Authors:** Xiangzhou Tan, Jianping Guo, Zihua Chen, Alfred Königsrainer, Dörte Wichmann

**Affiliations:** Department of General Surgery, Xiangya Hospital, Central South University, Changsha, China; Department of General, Visceral and Transplantation Surgery, Interdisciplinary Endoscopy Unit, University Hospital Tübingen, Tübingen, Germany; Department of General Surgery, Xiangya Hospital, Central South University, Changsha, China; Department of General Surgery, Xiangya Hospital, Central South University, Changsha, China; Department of General, Visceral and Transplantation Surgery, Interdisciplinary Endoscopy Unit, University Hospital Tübingen, Tübingen, Germany; Department of General, Visceral and Transplantation Surgery, Interdisciplinary Endoscopy Unit, University Hospital Tübingen, Hoppe-Seyler-Str. 3, 72076 Tübingen, Germany

**Keywords:** COVID-19, endoscopy, SARS-CoV-2 infection

## Abstract

**Background:**

The impact of gastrointestinal endoscopy on COVID-19 infection remains poorly investigated. We herein performed a systematic review and meta-analysis to evaluate the outcomes of COVID-19 in patients undergoing gastrointestinal endoscopy.

**Method:**

Ovid Medline, Ovid EMBASE, Ovid the Cochrane Library, and other electronic databases were searched until 30 November 2020 to identify publications with confirmed COVID-19 infection in patients undergoing gastrointestinal endoscopy. The primary outcomes were SARS-CoV-2 transmission, personal protective equipment use, rates of case fatality, complications, and procedural success.

**Results:**

A total of 18 articles involving 329 patients were included in this systematic review and meta-analysis. The overall basic reproduction rate is 0.37, while the subgroup results from Asia, Europe, and North America are 0.13, 0.44, and 0.33, respectively. The differences in personal protective equipment use between the positive transmission and non-transmission group are mainly in isolation gowns, N95 or equivalent masks, and goggles or face-shields. The rate of case fatality, complication, and procedural success are 0.17 (95% confidence interval = 0.02–0.38), 0.00 (95% confidence interval = 0.00–0.02), and 0.89 (95% confidence interval = 0.50–1.00), respectively. The fatality rate in Europe was the highest (0.23, 95% confidence interval = 0.04–0.50), which is significantly different from other continents (*p* = 0.034).

**Conclusion:**

The risk of SARS-CoV-2 transmission within gastrointestinal endoscopy units is considerably low if proper use of personal protective equipment is applied. Similarly, a low fatality and complication rate, as well as a high procedural success rate, indicated that a full recovery of endoscopic units should be considered.

## Introduction

The outbreak of the novel coronavirus disease 2019 (COVID-19), caused by SARS-CoV2 infections, has widely spread throughout the world^1,2^ and has been declared as a pandemic by the World Health Organization (WHO) in March 2020.^3^ So far, more than 119 million confirmed COVID-19 cases have been reported, with 2.6 million confirmed death by 17 March 2021.^4^ COVID-19 is not only able to cause respiratory illness, but may also lead to gastrointestinal (GI) diseases, for example, enteritis,^5,6^ pancreatitis,^7^ and cholangitis.^8^ The potential mechanism might be that SARS-CoV2 binds to the angiotensin-converting enzyme-2 (ACE2) receptors, which are widely expressed in lung, intestine, and liver.^9,10^ Hence, COVID-19 may have significant impact on the clinical outcomes of GI endoscopic procedures, including disease transmission, rate of fatality, complications, and procedural success.

Up to date, the impact of patients with SARS-CoV-2 infections undergoing GI endoscopy is poorly understood, leading to a controversial discussion. For instance, endoscopic procedures are associated with aerosol generation, which may pose significant risks to health care workers (HCWs) and patients. Here, Repici and colleagues^11^ declared that there is a low risk of SARS-CoV-2 transmission within GI endoscopy units with an infection rate of 4.2%. Of note, the burden of SARS-CoV-2-positive patients in their study is significantly low (1 of 802 patients, 0.12%), which compromises the reliability of the conclusion. The SARS-CoV-2 transmission rates within GI endoscopy units remain unknown.^12^ Another example is the management of gastrointestinal bleeding (GIB). According to the study by Martin and colleagues,^13^ no significant difference in transfusion requirements was observed between the endoscopic therapy group and the expectant therapy group. Conservative management was regarded as a reasonable approach in managing even complex GIB cases. In contrast, Saibeni and colleagues^14^ reported on three hospitalized GIB patients who did not receive an endoscopic evaluation, and two of them finally died. The management of endoscopic procedures volume is also a matter of debate. Resumption of endoscopic procedures in a safe setting is of critical importance in regard to mitigate adverse health outcomes caused by the pandemic.^15^ It is explorable to finger out the role of procedural volume reduction on restarting endoscopy service. Therefore, a study to assess the outcomes of COVID-19 in patients undergoing GI endoscopic procedures is urgently needed.

This systematic review and meta-analysis aimed to report on clinical outcomes of COVID-19 patients who underwent GI endoscopy based on the available data within the identified articles. Here, special emphasis will be put on disease transmission, personal protective equipment (PPE) use, rates of case fatality, complications, and procedural success in COVID-positive patients within endoscopic units. The study may therefore provide evidence-based guidance for clinical decision-making.

## Methods

The systematic review and meta-analysis were conducted using the Preferred Reporting Items for Systematic Reviews and Meta-Analyses (PRISMA) guidelines. The protocol for the study was registered and approved on PROSPERO (CRD42020225000).

### Search strategy

The publications that published on several electronic databases, registries, and guideline, including Ovid Medline (1950-present), Ovid EMBASE (1974- present), Ovid the Cochrane Library of Randomized Trials (1993-present), the World Health Organization International Clinical Trials Platform Search Portal (ICTRP), and ClinicalTrials.gov, were searched on 15 November 2020, using the key terms ‘2019nCoV’, ‘novel corona virus’, ‘COVID19’, ‘betacoronavirus’, ‘SARS-CoV-2’, ‘coronavirus infection’, ‘Wuhan pneumonia’, AND ‘endoscopy’, ‘gastroscopy’, ‘duodenoscopy’, ‘choledochoscopy’, ‘cholangioscopy’, ‘colonoscopy’, ‘rectoscopy’, and ‘proctoscopy’. Detailed retrieval strategy is shown in Supplementary Table 1s.

### Study selection and data extraction

Two investigators (X.T. and J.G.) performed the literature screening independently, based on the predefined inclusion and exclusion criteria.

Inclusion criteria were as follows: (1) studies reported data on COVID-19-confirmed patients; (2) these patients underwent GI endoscopy for both diagnosis and therapy; (3) had one of the following outcomes: transmission of SARS-CoV-2, PPE use, fatality, complications, success rate of endoscopic hemostasis; (4) the latest study was included if duplicated studies from the same population were identified; and (5) studies limited to human.

Exclusion criteria include the following: (1) no useful data, for example, outcomes, can be obtained; (2) small sample-size studies (*n* ⩽ 3) reported COVID-19 fatality, complications, or success rate of endoscopic therapy; (c) no sample-size limit in studies that reported transmission of SARS-CoV-2; (d) duplicated studies; and (e) non-English publications.

In cases of discrepancy and disagreement, the studies were discussed and resolved by consensus, or, if necessary, a third reviewer (D.W.) would be involved.^16^

Data extraction was independently conducted by two reviewers (X.T. and J.G.). The following information from each included study was extracted: first author, publication date, country, continent, study design type, number of COVID-19-positive patients, number of total observed patients, endoscopic reasons, ages, sex, and outcomes (transmission, fatality, complications, success rate of endoscopic therapy). Additional data, such as intensive care unit (ICU) admission rate, intervention rate, and upper bleeding rate, was obtained in the studies that reported COVID-19 fatality, complications, or success rate of endoscopic interventions.

### Endpoint setting and stratification strategy

Our primary outcomes were transmission of SARS-CoV-2 within GI endoscopic units, PPE use, case fatality rate, complication rate, and procedural success rate in COVID-19-positive patients who have undergone GI endoscopy. The stratified analysis of subgroups in different continents was carried out to determine the differences in outcomes in diverse regions.

### Meta-regression and assessment of bias

The univariable meta-regression analysis was performed to predict the impact of patient populations’ characteristics on several outcomes, including rates of fatality, complications, and procedural success. The factors we adopted for the meta-regression analysis involve continents, study period, mean age, male proportion, ICU admission rate, upper GI bleeding rate, and endoscopic intervention rate. Two parameters, that is, continents and study period, are dichotomous data; therefore, studies of North America in continents set and studies started before 11 March 2020 in study period set (the date COVID-19 was announced by WHO to be characterized as a pandemic) were defined as reference studies.

Visual inspection of funnel plots, Egger’s regression asymmetry test, and Begg’s rank correlation test in each outcome were applied to detect publication bias (*p*-value < 0.10).^17^

### Statistical analysis

The pooled proportion data with their corresponding 95% confidence intervals (CIs) were calculated via either a fixed-effects model or random-effects model, using the Freeman–Tukey double arcsine transformation method. The *Q* test and Higgins *I*^2^ static were applied to assess the between-study heterogeneity. Conventionally, an *I*^2^ value > 50% or *p*-value  < 0.05 was considered as high heterogeneity. A random-effects model was used when significant heterogeneity was identified; otherwise, a fixed-effects model was preferred. A meta-regression permutation test was applied to calculate the exact *p*-value.^18^ All statistical analyses were conducted with STATA 14.0 (Stata Corporation, College Station, Texas, USA).

## Results

### Literature search

**Figure 1.**
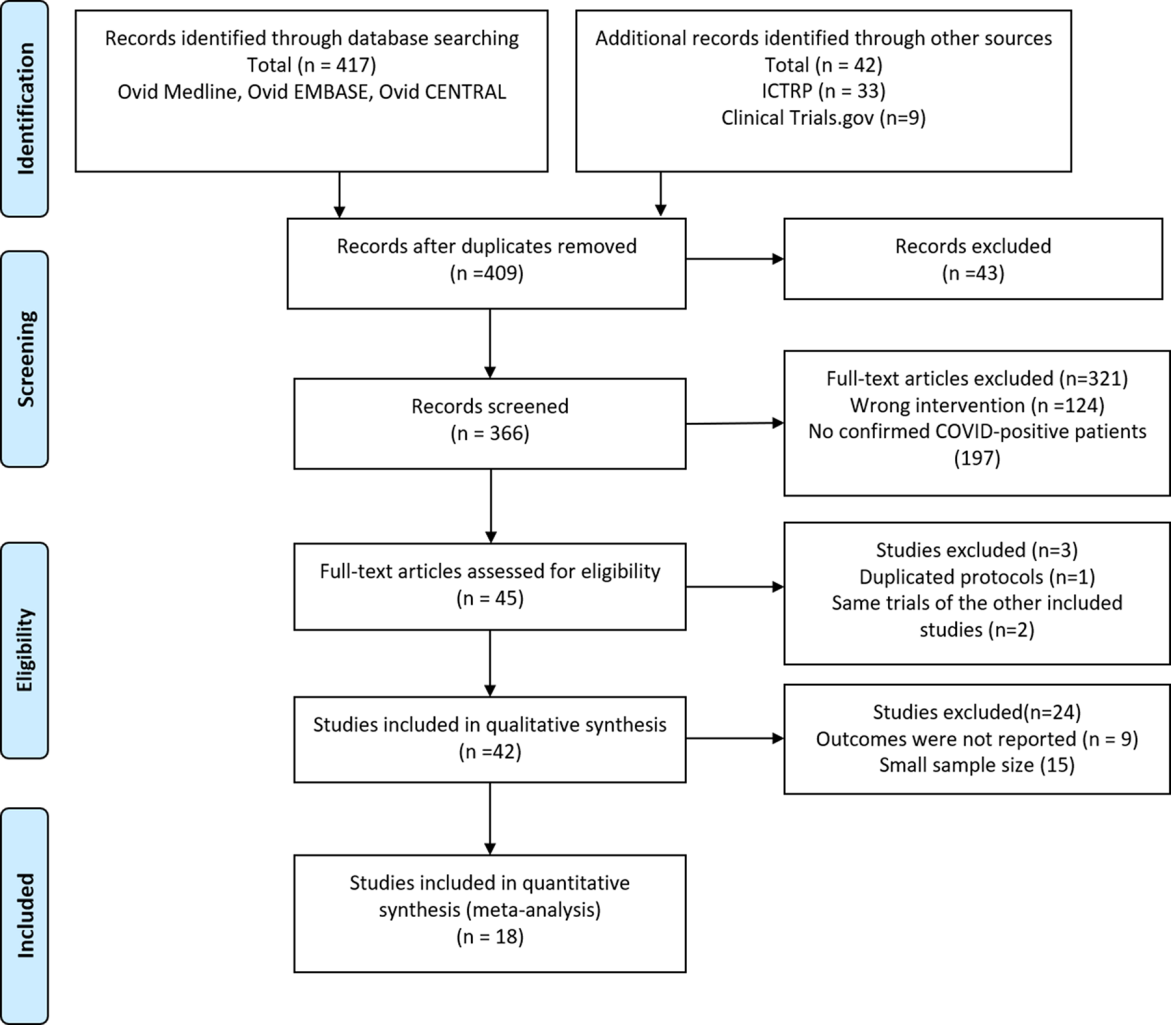
Flow chart of literature search (PRISMA 2009 flow diagram).

The initial search of databases identified 452 articles. After duplicate removal, a total of 409 studies were reviewed via title and abstract, which further lowered the number of studies to 366. Another 321 articles were then excluded, because they did not report the confirmed SARS-CoV-2-positive patients who were concurrently undergoing GI endoscopy. Another three studies were removed because the publication was about the same population as another included study. And 24 studies were excluded due to the limited outcomes data available and small sample size. Finally, 18 studies were included in the qualitative synthesis and the meta-analysis. The study selection flow diagram is shown in Figure 1.

### Study characteristics

A total of 18 studies involving 329 COVID-positive patients were included in the systematic review and meta-analysis. Baseline characteristics of included studies are listed in Table 1. There are eight studies (*n* = 150 patients) from Europe, six studies (*n* = 24 patients) from Asia, and four studies (*n* = 155 patients) from North America (all from the United States), respectively. No article has yet to be reported from Africa, South America, or Oceania. Three studies are descriptive studies, while 15 studies are other study types, including 2 retrospective cohort studies, 5 cross-sectional studies, and 8 case series or reports. The endoscopic indications involve GIB, emergency endoscopy, and others. The distribution of sex in the reported studies (*n* = 10) is 160 and 73 for numbers of male and female, respectively. However, there are eight studies which did not report the sex proportion.

**Table 1.** Characteristics of included studies.

Author	Counties	Continent	Study design	No. of COVID patients	No. of total patients	Endoscopy reasons	Age (years) mean ± SD/median (range)	Sex (M/F)
Podboy and colleagues^19^	United States	North America	Cross-sectional	2	1041	Not specific	NR	NR
Forde and colleagues^20^	United States	North America	Cross-sectional	1	396	Not specific	NR	NR
Martin and colleagues^13^	United States	North America	Cohort	41	123	GIB	NR	27/14
Wander and colleagues^21^	United States	North America	Case series	111	111	Emergency endoscopy	63.5 ± 16.74	75/36
Repici and colleagues^22^	Italy	Europe	Descriptive study	75	NR	Not specific	NR	NR
Repici and colleagues^11^	Italy	Europe	Descriptive study	1	802	Not specific	NR	NR
O’Grady and colleagues^23^	Ireland	Europe	Cross-sectional	1	55	Not specific	NR	NR
Lamazza and colleagues^24^	Italy	Europe	Cross-sectional	8	70	Not specific	NR	NR
Tavabie and colleagues^25^	United Kingdom	Europe	Cohort	19	203	UGI bleeds	60 (51–73)	14/5
Massironi and colleagues^26^	Italy	Europe	Case series	38	38	Not specific	71	28/10
Wichmann and colleagues^27^	Germany	Europe	Case series	7	7	GIB	62.4 ± 15.4	5/2
Dietrich and colleagues^28^	Germany	Europe	Case report	1	1	Inhomogeneous pancreatic tissue	72	1/-
Yu and colleagues^29^	China	Asia	Descriptive study	7	159	Emergency endoscopy	NR	NR
Kim and Kim^30^	Korea	Asia	Cross-sectional	1	130	Emergency endoscopy	NR	NR
Gu and colleagues^31^	China	Asia	Case series	12	12	Nutrition tube clogging, Tube dislocation, and GIB	72.8 (36–90)	8/4
Wang and colleagues^32^	China	Asia	Case series	2	2	Hematochezia	32;45	1/1
Zhai and colleagues^33^	China	Asia	Case report	1	1	Acute obstructive suppurative cholangitis	71	-/1
Kim and colleagues^34^	Korea	Asia	Case report	1	1	Follow-up check	48	1/-

GIB, gastrointestinal bleeding; NR, not reported; SD, standard deviation; UGI, upper gastrointestinal.

### Outcomes of COVID-19 patients who have undergone GI endoscopy

#### Transmission of SARS-CoV-2 and PPE use within GI endoscopy units

**Figure 2.**
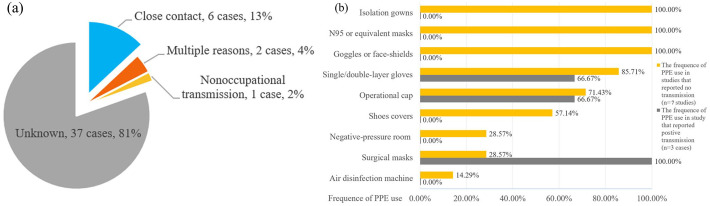
The reported COVID-19-positive cases transmitted within GI endoscopic unit and the presumed reasons (a), and the personal protective equipment (PPE) use presented in seven studies which reported no SARS-CoV-2 transmission within GI endoscopic unit. (b) Multiple reason refers to the combination of close contact and nonoccupational transmission.

Fifteen studies with a total of 122 COVID-positive patients reported the potential COVID-19 transmission rate, as shown in Table 2.^11,19,20,22–24,26–34^ Three studies^11,19,32^ reported potential transmission of SARS-CoV-2 within GI endoscopy units, for a total of 46 HCWs or patients. The total basic reproduction rate (R0) equals 0.37, while the subgroup results from Asia, Europe, and America are 0.13, 0.44, and 0.33, respectively. Although the transmission routes of most cases remain unknown, transmission via close contact with COVID-positive patients is the most common reason for traceable cases (6 out of 9 cases, 66.7%; see details in Figure 2(a)).

**Table 2.** The systematic review of COVID-19 transmissions within GI endoscopy units.

Studies	Counties	Publication date	Number of COVID-19-positive patients	Number of potential transmissions	Basic reproduction rate (R0)
Asia (6 studies)					
Gu and colleagues^31^	China	2020 May	12	0	0.00
Zhai and colleagues^33^	China	2020 November	1	0	0.00
Yu and colleagues^29^	China	2020 September	7	0	0.00
Wang and colleagues^32^	China	2020 November	1	3	3.00
Kim and colleagues^34^	South Korea	2020 November	1	0	0.00
Kim and Kim^30^	South Korea	2020 July	1	0	0.00
		Subgroup	23	3	0.13
Europe (7 studies)					
Wichmann and colleagues^27^	Germany	2020 November	7	0	0.00
Dietrich and colleagues^28^	Germany	2020 September	1	0	0.00
O’Grady and colleagues^23^	Ireland	2020 July	1	0	0.00
Massironi and colleagues^26^	Italy	2020 September	38	0	0.00
Repici and colleagues^22^	Italy	2020 April	40	42	1.05
Repici and colleagues^11^	Italy	2020 April	1	0	0.00
Lamazza and colleagues^24^	Italy	2020 July	8	0	0.00
		Subgroup	96	42	0.44
America (2 studies)					
Podboy and colleagues^19^	United States	2020 June	2	1	0.50
Forde and colleagues^20^	United States	2020 May	1	0	0.00
		Subgroup	3	1	0.33
In total		15 studies	122	46	0.37

GI, gastrointestinal.

The effect of PPE use on the transmission of SARS-CoV-2 within GI endoscopy units is evaluated by comparing the differences of PPE use between the studies that reported no transmission and the studies that reported positive transmissions. Eight studies^20,27,29–34^ were finally included for the analysis of PPE use in GI endoscopy units. Seven studies^20,27,29–31,33,34^ claimed that no transmission of SARS-CoV-2 was found in their GI endoscopy unit, whereas one study^32^ demonstrated successful screening of 3 COVID-positive transmitted cases. As is shown in Figure 2(b), the frequencies of PPE use in two group sets (no transmission group and positive transmissions group) were displayed. Three kinds of PPE were deemed to be the most important equipment for protecting HCWs from corona virus disease, including isolation gowns, N95 or equivalent masks, and goggles or face-shields.

#### Case fatality rate in COVID-positive patients undergoing GI endoscopy

**Figure 3.**
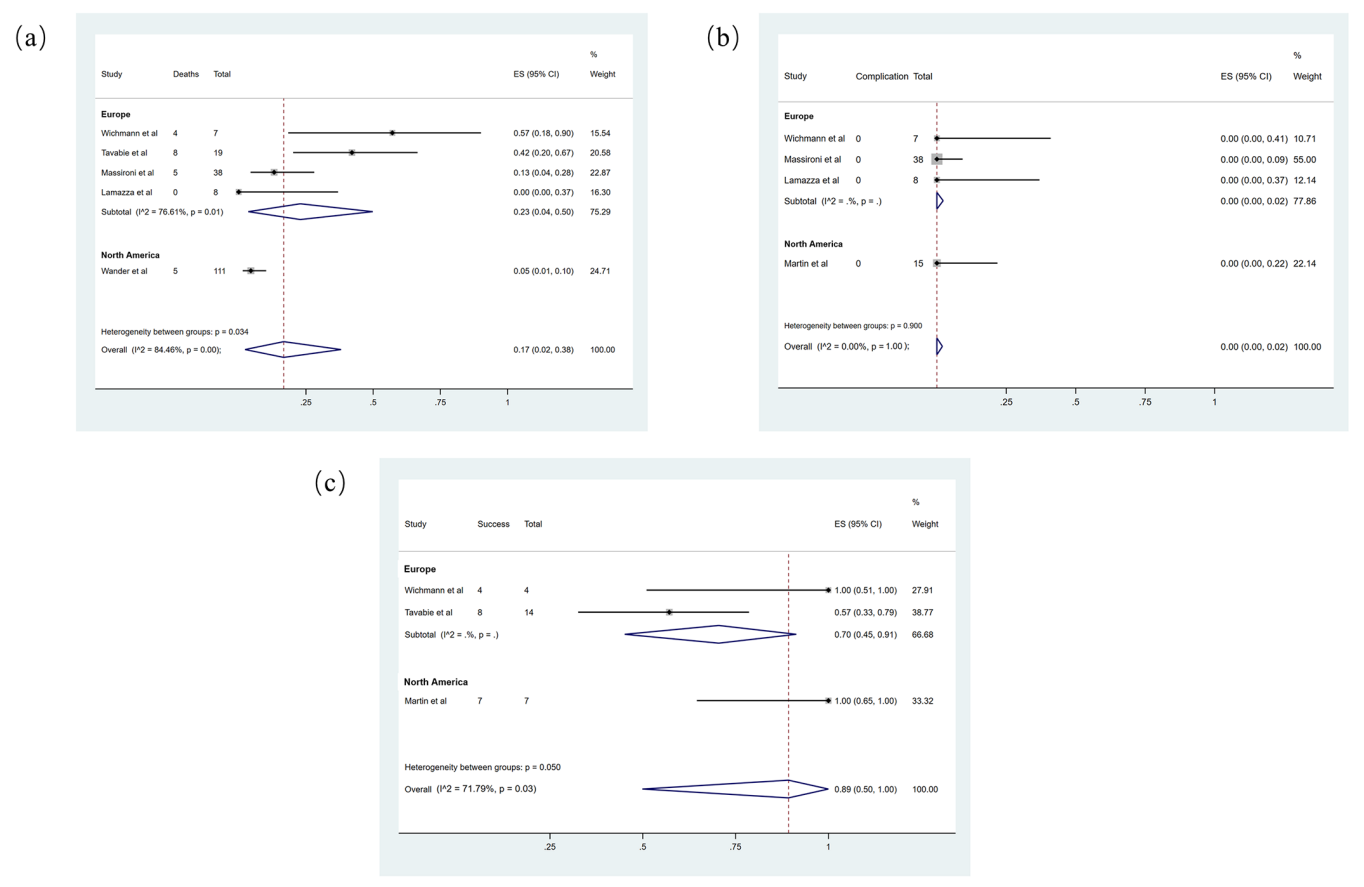
(a) Forest plot of case fatality rate, (b) forest plot of complication rate, and (c) forest plot of success rate in endoscopic hemostasis.

Five studies^21,24–27^ with a total of 183 COVID-positive subjects reported the case fatality rate in confirmed COVID-19 patients undergoing GI endoscopy (Figure 3(a)). Random-effects meta-analysis demonstrates that the total case fatality rate is 0.17 (95% CI = 0.02–0.38). Subgroup analysis among different continents shows significant differences between populations in Europe compared with North America. The case fatality rate in Europe [0.23 (95% CI = 0.04–0.50)] is significantly higher than the one reported from North America [0.05 (95% CI = 0.01–0.10)]. No studies from Asia reported the case fatality rate in COVID-positive patients undergoing GI endoscopy. Furthermore, high heterogeneity across all studies was observed (*I*^2^ = 84.46%).

#### Complication rate in COVID-positive patients who have undergone GI endoscopy

Four studies^13,24–27^ with a total of 68 COVID-positive patients pointed out the complication rate in confirmed COVID-19 patients who have undergone GI endoscopy (Figure 3(b)). The pooled complication rate is 0.00 (95% CI = 0.00–0.02), and there is no statistically significant difference between the European group and the North American Group (*p*-value = 0.90) using a fixed analysis. Only one study is available from North American for the subgroup analysis. Again, no Asian study reported the complication rate in the investigated population. There is no significant heterogeneity between included studies (*I*^2^ = 0.00%).

#### Procedural success rate in COVID-positive patients who have undergone GI endoscopy

In total, 41 subjects from 3 studies^13,25,27^ are included in the quantitative analysis of procedural success rate (Figure 3(c)). Only the outcome data from endoscopic hemostasis procedures can be obtained. The pooled procedural success rate equals to 0.89 (95% CI = 0.50–1.00) using random-effects models (*I*^2^ = 71.79%). Subgroup analysis shows no significant difference is observed between subgroups (*p*-value = 0.05).

### Meta-regression

A univariate meta-regression analysis was conducted to evaluate the effects of populations’ characteristics on each outcome. The variables that potentially influence outcomes, such as continents, study periods, mean age, male proportion, ICU admission rate, upper GI bleeding rate, and endoscopic intervention rate, were included for the meta-regression analysis. The results of case fatality rate, complication rate, and procedural success rate show that none of the included factors is significantly different to affect Case Fatality Rate (CFR) (Table 3). However, ICU admission rate is a variable that contributes to the complication rate of COVID-positive patients undergoing GI endoscopy (*p*-value = 0.041, SE = 0.006).

**Table 3.** Meta-regression of case fatality rate, complication rate, and endoscopic hemostasis.

Factors	Fatality rate		Complication rate		Procedural success rate	
	Studies (*n*)	*p*-value	Studies (*n*)	*p*-value	Studies (*n*)	*p*-value
Continents	5	0.922	4	1.000	3	1.000
Study period	5	0.204	4	1.000	3	0.348
Age	4	0.992	3	1.000	3	0.856
Male proportion (%)	4	0.716	3	1.000	3	0.668
ICU admission (%)	4	0.117	3	1.000	3	0.402
Upper GI bleeding (%)	3	0.764	3	1.000	3	0.163

GI, gastrointestinal; ICU, intensive care unit.

### Assessment of publication bias

Visual inspection of funnel plots and Egger’s plots are applied to assess the publication bias. The funnel plot and Egger’s plots of studies on each outcome is illustrated in Supplementary Figure 1s and Figure 2s, respectively. No evidence of obvious asymmetry is found in the funnel plots. The statistical results of both Egger’s and Begg’s test support there is no publication bias in our study (Egger’s test: *p*-value = 0.109 for case fatality rate, *p*-value = 1.000 for complication rate, and *p*-value = 0.758 for procedural success rate; Begg’s test: *p*-value = 0.462 for case fatality rate, *p*-value = 1.000 for complication rate, and *p*-value = 1.000 for procedural success rate).

## Discussion

We conduct the first systematic review and meta-analysis to explore the clinical outcomes of COVID-19 patients within GI endoscopy units, including 329 patients from 18 studies worldwide. Overall, the basic reproduction rate (R0) for SARS-CoV-2 transmission within GI endoscopic units was 0.37, which is significantly lower than the reported R0 with a range from 2 to 4.^35^ The rate of case fatality, complications, and procedural success among SARS-CoV-2-infected patients undergoing GI endoscopy was 0.17 (95% CI = 0.04–0.35), 0.00 (95% CI = 0.00–0.02), and 0.89 (95% CI = 0.50–1.00), respectively. These results imply that under the sufficient supply of PPE, GI endoscopy is a safe and efficient approach for the diagnosis and treatment of digestive diseases in COVID-19-positive patients. Emergency endoscopy should be fully considered irrespective of SARS-CoV2 infection status. In addition, elective GI endoscopy is supposed to return to pre-pandemic volumes after the COVID-19 pandemic has declined.

We noted that the cross-study heterogeneity is considerably high. Most cases were reported from Europe with a higher fatality rate, complication rate, and lower procedural success rate. Although the subgroup and meta-regression analysis were performed, the source of heterogeneity could not be fully explained. Here, we assume that multiple reasons may lead to the high heterogeneity among different studies. First, the inclusion criteria of GI endoscopy for COVID-positive patients vary significantly between the studies included. The indications for endoscopy could be emergency endoscopy, GIB, dealing with the complications of nutrition tube (e.g. dislocation or clogging), which is also influenced by local epidemic conditions, implemented policies, and medical supplies. Second, there are no large-scale data of COVID-19 infections patients within GI endoscopy units owing to the reduction of endoscopy volume to minimize the transmission of SARS-CoV-2. The baseline characteristics of included patients, including age, sex proportion, and ICU admission rate, are therefore quite different. Third, as the continuous mutation of SARS-CoV-2, the individual genotypes of virus may also contribute to the differences in clinical outcomes. Recently, SARS-CoV-2 variant B.1.1.7, initially detected in the United Kingdom, was proved to be associated with a higher transmission risk, severities, and fatality of the disease.^36^

Our study systematically reviewed the available data on transmission of SARS-CoV-2 within GI endoscopy units and provided solid evidence for a considerable low risk of SARS-CoV2 infections for both HCWs and patients. Almost 50% of COVID-19-positive patients have shown detectable virus RNA in their fecal samples.^37,38^ Therefore, there are considerable concerns arising about the potential fecal-oral or fecal-aerosol transmission, which imposes GI endoscopic experts to implement strict precautions, such as a high threshold for admitting endoscopy, to minimize the infection risk during GI endoscopy, especially for aerosol-generating procedures (AGP). However, our results demonstrated there is very limited transmission of SARS-CoV-2 in GI endoscopic units if proper PPE was applied. Close contact is still the predominant transmission route. A proper PPE should at least include isolation gowns, N95 or equivalent masks, and goggles or face-shields. Of note, the study by Wang and colleagues demonstrated that wearing a surgical mask during GI endoscopy is insufficient for preventing transmission of SARS-CoV-2,^32^ which is consistent with other studies.^39^ Interestingly, Kim and colleagues^34^ reported about a patient with unknown COVID-infection at time of examination who wore a surgical mask in a GI endoscopy unit: no patients in the recovery room nor HCWs were infected. This case report suggested that precautions from patients’ side could also provide an efficient approach to lower the risk of COVID transmission, but the possibility of individual transmission capability should also be taken into account.

In our results, the fatality rate of COVID-19 patients undergoing GI endoscopy (0.17, 95% CI = 0.04–0.35) seems to be relatively high, compared with the CFR around 2% that had been reported by the WHO.^4^ However, nearly all cases that had been included in the investigation of CFR were from hospitalized populations, part of them are even from ICUs. This phenomenon is mostly assignable to a high threshold of endoscopy admission that bases on expert consensus.^40^ The mortality of in-hospital patients with COVID-19 was reported at 28–39% and even higher in patients with comorbidities.^41–45^ These data significantly exceed the CFR in our study. In addition, those patients with hemodynamic instability, emergent bleeding, obstructive jaundice, and so on are more likely to be referred to endoscopic units.^46^ These symptoms normally accompany with high risk of fatality. Several studies also revealed that most cases did not die of endoscopy-related incidents but due to SARS-CoV2 infections.^21,25–27^ Hence, we believe the high CFR of COVID-19 patients within GI endoscopy units is dependent of the characteristic baseline of included population. Hence, we strongly believe that the endoscopy-related CFR of COVID-19 patients who receive GI endoscopy is significantly low.

When analyzing complications, the systematic review and meta-analysis demonstrate a low complication rate in COVID-19 patients undergoing GI endoscopy (0.00, 95% CI = 0.00–0.02). In the current publications, there are no major complications associated with GI endoscopic procedures. The low complication rates, in some extent, indirectly support our hypothesis of low endoscopy-related CFR. In regard to endoscopic intervention, only the data involving endoscopic hemostasis can be obtained; the success rate of endoscopic hemostasis was therefore analyzed based on three studies.^13,25,27^ The endoscopic hemostasis procedure shows a high success rate with a pooled value of 89% (95% CI = 50%–100%). The main interventions for hemostasis are endoscopic clipping, hemostatic injection, rectal packing, cautery, and so on.

It has been reported that the risk of death from SARS-CoV2 infections in COVID-19 patients who have undergone treatment for other comorbidities, such as cancer, was overestimated.^47^ A recent meta-analysis and a large European cohort study pointed out that insufficient cancer treatments play a role in the fatality and severity of COVID-19.^48,49^ Our results proved a low risk of transmission, endoscopy-related case fatality, complication, and procedural failure in COVID-19 patients who received GI endoscopy. The endoscopic diagnosis and treatments for comorbidities (e.g. cancer), apart from SARS-CoV-2 infections, are highly recommended to be continued during the era of epidemics.

A few methodological limitations existed in this study. First, most included publications are small-sample case series or cohort studies. Second, the inclusion and exclusion criteria of admitting endoscopy for COVID-19 patients are different in various endoscopic units due to medical supplies, local anti-epidemic policy, and healthcare burden. Finally, the overall heterogeneity is significantly high and could not be fully explained by subgroup and meta-regression analysis, which probably undermines the reliability of our conclusion. However, few cases of data can be recorded because of the rigorous restriction of expert consensus. This systematic review and meta-analysis comprehensively collected and analyzed the clinical outcomes of COVID-19 patients receiving GI endoscopy, which can guide the clinical practices for reopening GI endoscopy.

## Conclusion

Many endoscopy units recently resume routine procedures to catch up on the postponed cases during COVID-19 pandemics. The clinical outcomes of COVID-19 patients receiving endoscopy are of significant importance. Our study demonstrates a low risk of SARS-CoV-2 transmission within GI endoscopy. The analysis of the PPE use suggests appropriate equipment was recommended to include isolation gowns, N95 or equivalent masks, and goggles or face-shields. In addition, a low rate of fatality, complication, and a high procedural success rate exhibit the need to lift the restriction for admission of GI endoscopy. Our results may provide evidence-based recommendations for the resumption of GI endoscopy.

## Supplemental Material

sj-docx-3-tag-10.1177_17562848211042185 – Supplemental material for Systematic review and meta-analysis of clinical outcomes of COVID-19 patients undergoing gastrointestinal endoscopy Supplemental material, sj-docx-3-tag-10.1177_17562848211042185 for Systematic review and meta-analysis of clinical outcomes of COVID-19 patients undergoing gastrointestinal endoscopy by Xiangzhou Tan, Jianping Guo, Zihua Chen, Alfred Königsrainer and Dörte Wichmann in Therapeutic Advances in Gastroenterology

sj-tif-1-tag-10.1177_17562848211042185 – Supplemental material for Systematic review and meta-analysis of clinical outcomes of COVID-19 patients undergoing gastrointestinal endoscopy Supplemental material, sj-tif-1-tag-10.1177_17562848211042185 for Systematic review and meta-analysis of clinical outcomes of COVID-19 patients undergoing gastrointestinal endoscopy by Xiangzhou Tan, Jianping Guo, Zihua Chen, Alfred Königsrainer and Dörte Wichmann in Therapeutic Advances in Gastroenterology

sj-tif-2-tag-10.1177_17562848211042185 – Supplemental material for Systematic review and meta-analysis of clinical outcomes of COVID-19 patients undergoing gastrointestinal endoscopy Supplemental material, sj-tif-2-tag-10.1177_17562848211042185 for Systematic review and meta-analysis of clinical outcomes of COVID-19 patients undergoing gastrointestinal endoscopy by Xiangzhou Tan, Jianping Guo, Zihua Chen, Alfred Königsrainer and Dörte Wichmann in Therapeutic Advances in Gastroenterology

## References

[1] Guan WJ Ni ZY Hu Y , et al. Clinical characteristics of coronavirus disease 2019 in China. N Engl J Med 2020; 382: 1708–1720. 32109013 10.1056/NEJMoa2002032 PMC7092819

[2] Huang C Wang Y Li X , et al. Clinical features of patients infected with 2019 novel coronavirus in Wuhan, China. Lancet 2020; 395: 497–506. 31986264 10.1016/S0140-6736(20)30183-5 PMC7159299

[3] WHO. Archived: WHO timeline – COVID-19, https://www.who.int/news/item/27-04-2020-who-timeline—covid-19 (2020, accessed 17 March 2021).

[4] WHO. Coronavirus disease (COVID-19) pandemic view dashboard, https://www.who.int/emergencies/diseases/novel-coronavirus-2019 (2021, accessed 17 March 2021).

[5] Carvalho A Alqusairi R Adams A , et al. SARS-CoV-2 gastrointestinal infection causing hemorrhagic colitis: implications for detection and transmission of COVID-19 disease. Am J Gastroenterol 2020; 115: 942–946. 32496741 10.14309/ajg.0000000000000667 PMC7172485

[6] Zhou J Li C Liu X , et al. Infection of bat and human intestinal organoids by SARS-CoV-2. Nat Med 2020; 26: 1077–1083. 32405028 10.1038/s41591-020-0912-6

[7] Miao Y Lidove O Mauhin W. First case of acute pancreatitis related to SARS-CoV-2 infection. Br J Surg 2020; 107: e270. 10.1002/bjs.11741 PMC7300914 32492174

[8] Bartoli A Gitto S Sighinolfi P , et al. Primary biliary cholangitis associated with SARS-CoV2 infection. J Hepatol 2021; 74: 1245–1246. 33610679 10.1016/j.jhep.2021.02.006 PMC7892314

[9] Siddique SM Sultan S Lim JK , et al. Spotlight: COVID-19 PPE and endoscopy. Gastroenterology 2020; 159: 759. 32590006 10.1053/j.gastro.2020.06.047 PMC7309779

[10] Zippi M Fiorino S Occhigrossi G , et al. Hypertransaminasemia in the course of infection with SARS-CoV-2: incidence and pathogenetic hypothesis. World J Clin Cases 2020; 8: 1385–1390. 32368531 10.12998/wjcc.v8.i8.1385 PMC7190951

[11] Repici A Aragona G Cengia G , et al. Low risk of COVID-19 transmission in GI endoscopy. Gut 2020; 69: 1925–1927. 32321857 10.1136/gutjnl-2020-321341

[12] Corral JE Hoogenboom SA Kroner PT , et al. COVID-19 polymerase chain reaction testing before endoscopy: an economic analysis. Gastrointest Endosc 2020; 92: 524–534.e526. 10.1016/j.gie.2020.04.049 PMC7187877 32360302

[13] Martin TA Wan DW Hajifathalian K , et al. Gastrointestinal bleeding in patients with coronavirus disease 2019: a matched case-control study. Am J Gastroenterol 2020; 115: 1609–1616. 32796176 10.14309/ajg.0000000000000805 PMC7446989

[14] Saibeni S Scucchi L Dragoni G , et al. Activities related to inflammatory bowel disease management during and after the coronavirus disease 2019 lockdown in Italy: how to maintain standards of care. United European Gastroenterol J 2020; 8: 1228–1235. 10.1177/2050640620964132 PMC7724532 33070758

[15] Bhandari P Subramaniam S Bourke MJ , et al. Recovery of endoscopy services in the era of COVID-19: recommendations from an international Delphi consensus. Gut 2020; 69: 1915–1924. 32816921 10.1136/gutjnl-2020-322329

[16] Tan X Yang W Wichmann D , et al. Magnetic endoscopic imaging as a rational investment for specific colonoscopies: a systematic review and meta-analysis. Expert Rev Gastroenterol Hepatol 2021; 15: 447–458. 33267703 10.1080/17474124.2021.1842192

[17] Tan X Wen Q Wang R , et al. Chemotherapy-induced neutropenia and the prognosis of colorectal cancer: a meta-analysis of cohort studies. Expert Rev Anticancer Ther 2017; 17: 1077–1085. 28910204 10.1080/14737140.2017.1380521

[18] Phipson B Smyth GK. Permutation P-values should never be zero: calculating exact P-values when permutations are randomly drawn. Stat Appl Genet Mol Biol 2010; 9: 39. 10.2202/1544-6115.1585 21044043

[19] Podboy A Cholankeril G Cianfichi L , et al. Implementation and impact of universal preprocedure testing of patients for COVID-19 before endoscopy. Gastroenterology 2020; 159: 1586–1588.e1584. 10.1053/j.gastro.2020.06.022 PMC7833582 32562723

[20] Forde JJ Goldberg D Sussman D , et al. Yield and implications of pre-procedural COVID-19 polymerase chain reaction testing on routine endoscopic practice. Gastroenterology 2020; 159: 1538–1540. 32464146 10.1053/j.gastro.2020.05.062 PMC7255132

[21] Wander P Hogan DE Inamdar S , et al. Elective endotracheal intubation for urgent gastrointestinal endoscopy among hospitalized patients with SARS-CoV-2. Gastrointest Endosc 2020; 92: 992–995. 32565189 10.1016/j.gie.2020.06.039 PMC7301808

[22] Repici A Pace F Gabbiadini R , et al. Endoscopy units and the coronavirus disease 2019 outbreak: a multicenter experience from Italy. Gastroenterology 2020; 159: 363–366. 32283102 10.1053/j.gastro.2020.04.003 PMC7151374

[23] O’Grady J Leyden J MacMathuna P , et al. ERCP and SARS-COV-2: an urgent procedure that should be immune. Scand J Gastroenterol 2020; 55: 976–978. 32643467 10.1080/00365521.2020.1789210

[24] Lamazza A Fiori E Carati MV , et al. Therapeutic options for emergency gastrointestinal malignancy in COVID19 pandemic. Br J Surg 2020; 107: e403–e404. 10.1002/bjs.11846 PMC7929232 33448343

[25] Tavabie OD Clough JN Blackwell J , et al. Reduced survival after upper gastrointestinal bleed endoscopy in the COVID-19 era is a secondary effect of the response to the global pandemic: a retrospective cohort study. Frontline Gastroenterol 2020; 12: 279–287. 34249312 10.1136/flgastro-2020-101592 PMC8231434

[26] Massironi S Vigano C Dioscoridi L , et al. Endoscopic findings in patients infected with 2019 novel coronavirus in Lombardy, Italy. Clin Gastroenterol Hepatol 2020; 18: 2375–2377. 32480008 10.1016/j.cgh.2020.05.045 PMC7260560

[27] Wichmann D Atique NB Stuker D , et al. Impact of the COVID-19 pandemic on an interdisciplinary endoscopy unit in a German ‘hotspot’ area: a single center experience. Surg Endosc. Epub ahead of print 2 November 2020. DOI: 10.1007/s00464-020-08119-w. PMC7605334 33140149

[28] Dietrich CG Hubner D Marx G , et al. Primary presentation of COVID-19 solely with gastrointestinal symptoms: a problem for the containment of the disease. Eur J Gastroenterol Hepatol 2020; 32: 1475–1478. 32925503 10.1097/MEG.0000000000001922

[29] Yu Q Xu P Gan H , et al. Comprehensive gastroenterology endoscopy unit workflow and infection prevention during the COVID-19 pandemic: experience with 159 cases in Wuhan, China. Dig Endosc 2021; 33: 195–202. 32886846 10.1111/den.13832

[30] Kim SB Kim KH. The proposed algorithm for emergency endoscopy during the coronavirus disease 2019 outbreak. Korean J Intern Med 2020; 35: 1027–1030. 32664710 10.3904/kjim.2020.229 PMC7487316

[31] Gu Q Wang HF Fang Y , et al. Analysis of an improved workflow of endoscope reprocessing for bedside endoscopic diagnosis and treatment on COVID-19 patients. J Zhejiang Univ Sci B 2020; 21: 416–422. 32425010 10.1631/jzus.B2000109 PMC7210095

[32] Wang G Guan JL Zhu XQ , et al. Infection, screening, and psychological stress of health care workers with COVID-19 in a non-frontline clinical department. Disaster Med Public Health Prep. Epub ahead of print 4 November 2020. DOI: 10.1017/dmp.2020.428. PMC7884665 33143814

[33] Zhai LL Xiang F Wang W , et al. Atypical presentations of coronavirus disease 2019 in a patient with acute obstructive suppurative cholangitis. Clin Res Hepatol Gastroenterol 2020; 44: e135–e140. 32482542 10.1016/j.clinre.2020.05.003 PMC7241319

[34] Kim HJ Kwon YH Jeon SW , et al. Unexpected exposure to coronavirus disease at the endoscopic room: what should we do? Korean J Helicobacter Up Gastrointest Res 2020; 20: 248–250.

[35] Liu Y Gayle AA Wilder-Smith A , et al. The reproductive number of COVID-19 is higher compared to SARS coronavirus. J Travel Med 2020; 27: taaa021. 10.1093/jtm/taaa021 PMC7074654 32052846

[36] Davies NG Jarvis CI Edmunds WJ , et al. Increased hazard of death in community-tested cases of SARS-CoV-2 variant of concern 202012/01. medRxiv 2021, https://www.medrxiv.org/content/10.1101/2021.02.01.21250959v3

[37] Nobel YR Phipps M Zucker J , et al. Gastrointestinal symptoms and coronavirus disease 2019: a case-control study from the United States. Gastroenterology 2020; 159: 373–375.e372. 10.1053/j.gastro.2020.04.017 PMC7152871 32294477

[38] Cheung KS Hung IFN Chan PPY , et al. Gastrointestinal manifestations of SARS-CoV-2 infection and virus load in fecal samples from a Hong Kong cohort: systematic review and meta-analysis. Gastroenterology 2020; 159: 81–95. 32251668 10.1053/j.gastro.2020.03.065 PMC7194936

[39] Ng K Poon BH Kiat Puar TH , et al. COVID-19 and the risk to health care workers: a case report. Ann Intern Med 2020; 172: 766–767. 32176257 10.7326/L20-0175 PMC7081171

[40] Castro Filho EC Castro R Fernandes FF , et al. Gastrointestinal endoscopy during the COVID-19 pandemic: an updated review of guidelines and statements from international and national societies. Gastrointest Endosc 2020; 92: 440–445.e446. 10.1016/j.gie.2020.03.3854 PMC7130800 32268135

[41] Zhou F Yu T Du R , et al. Clinical course and risk factors for mortality of adult inpatients with COVID-19 in Wuhan, China: a retrospective cohort study. Lancet 2020; 395: 1054–1062. 32171076 10.1016/S0140-6736(20)30566-3 PMC7270627

[42] Cummings MJ Baldwin MR Abrams D , et al. Epidemiology, clinical course, and outcomes of critically ill adults with COVID-19 in New York City: a prospective cohort study. Lancet 2020; 395: 1763–1770. 32442528 10.1016/S0140-6736(20)31189-2 PMC7237188

[43] Docherty AB Harrison EM Green CA , et al. Features of 20 133 UK patients in hospital with covid-19 using the ISARIC WHO Clinical Characterisation Protocol: prospective observational cohort study. BMJ 2020; 369: m1985. 10.1136/bmj.m1985 PMC7243036 32444460

[44] Cheng Y Luo R Wang K , et al. Kidney disease is associated with in-hospital death of patients with COVID-19. Kidney Int 2020; 97: 829–838. 32247631 10.1016/j.kint.2020.03.005 PMC7110296

[45] Moon AM Webb GJ Aloman C , et al. High mortality rates for SARS-CoV-2 infection in patients with pre-existing chronic liver disease and cirrhosis: preliminary results from an international registry. J Hepatol 2020; 73: 705–708. 32446714 10.1016/j.jhep.2020.05.013 PMC7241346

[46] Gralnek IM Hassan C Beilenhoff U , et al. ESGE and ESGENA position statement on gastrointestinal endoscopy and the COVID-19 pandemic. Endoscopy 2020; 52: 483–490. 32303090 10.1055/a-1155-6229 PMC7295280

[47] Di Fiore F Bouche O Lepage C , et al. COVID-19 epidemic: proposed alternatives in the management of digestive cancers: a French intergroup clinical point of view (SNFGE, FFCD, GERCOR, UNICANCER, SFCD, SFED, SFRO, SFR). Dig Liver Dis 2020; 52: 597–603. 32418773 10.1016/j.dld.2020.03.031 PMC7255323

[48] Zhang H Han H He T , et al. Clinical characteristics and outcomes of COVID-19-infected cancer patients: a systematic review and meta-analysis. J Natl Cancer Inst 2021; 113: 371–380. 33136163 10.1093/jnci/djaa168 PMC7665647

[49] Lievre A Turpin A Ray-Coquard I , et al. Risk factors for coronavirus disease 2019 (COVID-19) severity and mortality among solid cancer patients and impact of the disease on anticancer treatment: a French Nationwide Cohort Study (GCO-002 CACOVID-19). Eur J Cancer 2020; 141: 62–81. 33129039 10.1016/j.ejca.2020.09.035 PMC7543792

