# Supplementary material for: Systematic review and meta-analysis of clinical outcomes of COVID-19 patients undergoing gastrointestinal endoscopy
Source: Ther Adv Gastroenterol. 2021 Aug 30;14:17562848211042185. doi: 10.1177/17562848211042185 (PMC8408897; doi:10.1177/17562848211042185)
Supplement: sj-docx-3-tag-10.1177_17562848211042185 – Supplemental material for Systematic review and meta-analysis of clinical outcomes of COVID-19 patients undergoing gastrointestinal endoscopy [file sj-docx-3-tag-10.1177_17562848211042185.docx]

Table s1 Search strategy

| The databases, including Ovid MEDLINE In-Process, Ovid EMBASE, Ovid Cochrane Database, and Scopus, were searched, from their inception to 15/11/2020. Only English articles were included. |
| --- |
| 1. (coronavir* OR corona virus* OR betacoronavir* OR covid19 OR covid 19 OR nCoV OR novel CoV OR CoV 2 OR CoV2 OR sarscov2 OR 2019nCoV OR wuhan virus*).mp. OR ((wuhan OR hubei OR huanan) AND (severe acute respiratory OR pneumonia*) AND outbreak*).mp. OR Coronavirus infection/ OR coronavirinae/ OR exp betacoronavirus/  Limits: 2020‐recent  2. (novel coronavir* OR novel corona virus* OR covid19 OR covid 19 OR nCoV OR novel CoV OR CoV 2 OR CoV2 OR sarscov2 OR 2019nCoV OR wuhan virus*).mp. OR ((wuhan OR hubei OR huanan) AND (severe acute respiratory OR pneumonia*) AND outbreak*).mp. OR ((wuhan OR hubei OR huanan) AND (coronavir* OR betacoronavir*)).mp.  Limits: 2019‐recent  3. or/1-2  4. exp endoscopy/  5. exp endoscopes/  6. endoscop* OR gastroscop* OR duodenoscop* colonoscop* OR choledochoscop* OR cholangioscop* OR rectoscop* OR proctoscop*  7. or/4-6  8. exp cohort analysis/  9. exp longitudinal study/  10. exp prospective study/  11. exp follow up/  12. cohort$.tw.  13. exp case control study/  14. (case$ and control$).tw.  15. exp case study/  16. (case$ and series).tw.  17. case report/  18. (case$ adj2 report$).tw.  19. (case$ adj2 stud$).tw.  20. or/8-19  21. #3 and #7 and #20  22. exp animals/ OR exp nonhuman/  23. exp humans/  24. #21 not (#22 not #23)  25 limit 24 to English language [Limit not valid in CDSR; records were retained]  26 remove duplicates from 25 |
