# Supplementary figures and images for: Systematic review and meta-analysis of clinical outcomes of COVID-19 patients undergoing gastrointestinal endoscopy
Source: Ther Adv Gastroenterol. 2021 Aug 30;14:17562848211042185. doi: 10.1177/17562848211042185 (PMC8408897; doi:10.1177/17562848211042185)

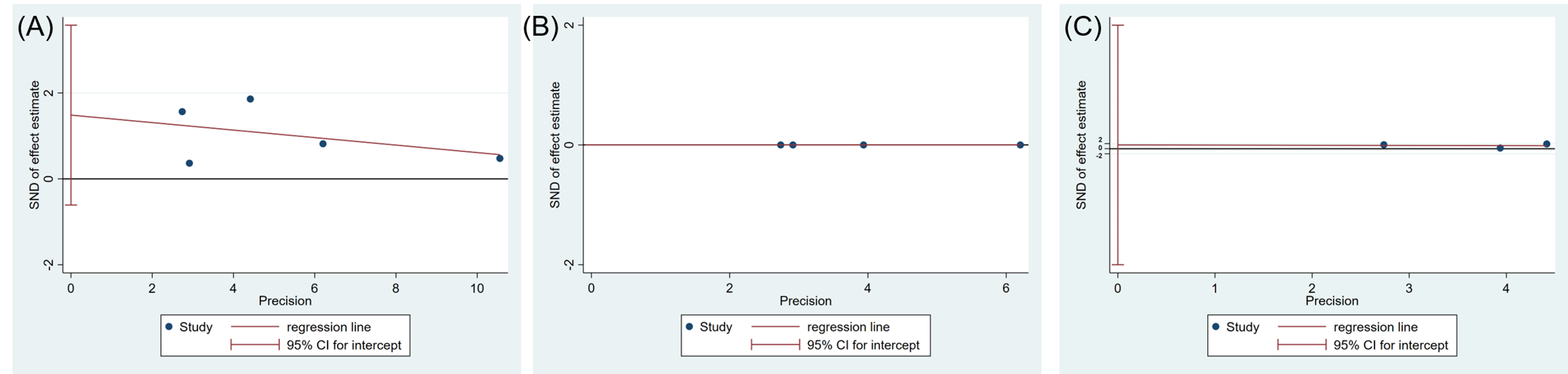

Supplement: sj-tif-1-tag-10.1177_17562848211042185 – Supplemental material for Systematic review and meta-analysis of clinical outcomes of COVID-19 patients undergoing gastrointestinal endoscopy [file sj-tif-1-tag-10.1177_17562848211042185.tif]

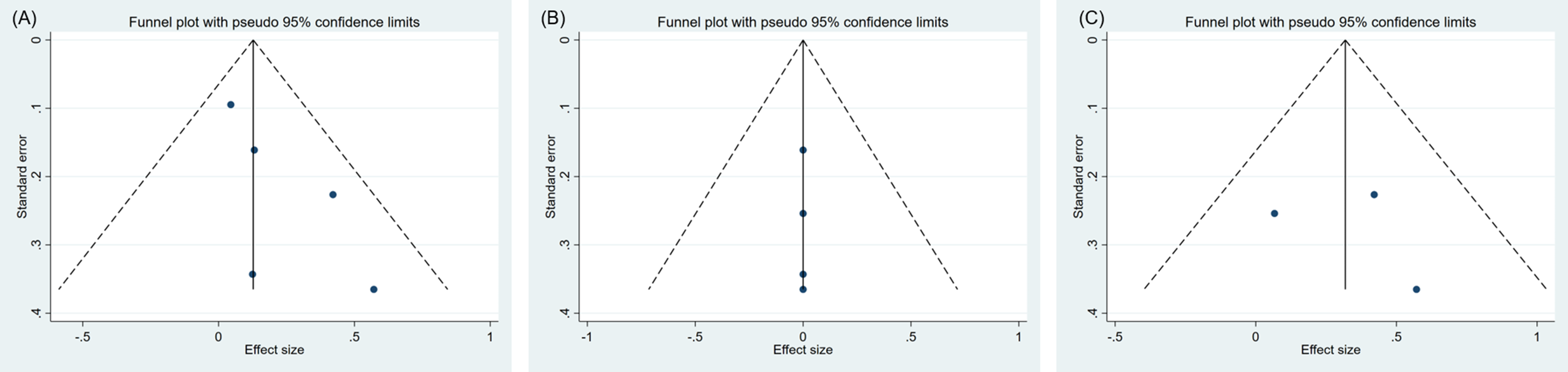

Supplement: sj-tif-2-tag-10.1177_17562848211042185 – Supplemental material for Systematic review and meta-analysis of clinical outcomes of COVID-19 patients undergoing gastrointestinal endoscopy [file sj-tif-2-tag-10.1177_17562848211042185.tif]
